# New Biomarkers in Liver Fibrosis: A Pass through the Quicksand?

**DOI:** 10.3390/jpm14080798

**Published:** 2024-07-29

**Authors:** Marzia Tagliaferro, Mariapaola Marino, Valerio Basile, Krizia Pocino, Gian Ludovico Rapaccini, Gabriele Ciasca, Umberto Basile, Valeria Carnazzo

**Affiliations:** 1 Dipartimento di Patologia Clinica, Ospedale Santa Maria Goretti, A.U.S.L. Latina, 04100 Latina, Italy; m.tagliaferro@ausl.latina.it (M.T.); v.carnazzo@ausl.latina.it (V.C.); 2 Dipartimento di Medicina e Chirurgia Traslazionale, Università Cattolica del Sacro Cuore, 00168 Rome, Italy; mariapaola.marino@unicatt.it (M.M.); gianludovico.rapaccini@unicatt.it (G.L.R.); 3 Fondazione Policlinico Universitario “A. Gemelli” IRCCS, Università Cattolica del Sacro Cuore, 00168 Rome, Italy; gabriele.ciasca@unicatt.it; 4 Clinical Pathology Unit and Cancer Biobank, Department of Research and Advanced Technologies, I.R.C.C.S. Regina Elena National Cancer Institute, 00144 Rome, Italy; valeriobasile90@gmail.com; 5 Clinical Pathology Unit, San Pietro Fatebenefratelli Hospital, 00189 Rome, Italy; pocino.krizia@fbfrm.it; 6 Dipartimento di Neuroscienze, Sezione di Fisica, Università Cattolica del Sacro Cuore, 00168 Rome, Italy

**Keywords:** liver fibrosis, biomarkers, extracellular matrix components

## Abstract

Chronic liver diseases (CLD) stem from various causes and lead to a gradual progression that ultimately may result in fibrosis and eventually cirrhosis. This process is typically prolonged and asymptomatic, characterized by the complex interplay among various cell types, signaling pathways, extracellular matrix components, and immune responses. With the prevalence of CLD increasing, diagnoses are often delayed, which leads to poor prognoses and in some cases, the need for liver transplants. Consequently, there is an urgent need for the development of novel, non-invasive methods for the diagnosis and monitoring of CLD. In this context, serum biomarkers—safer, repeatable, and more acceptable alternatives to tissue biopsies—are attracting significant research interest, although their clinical implementation is not yet widespread. This review summarizes the latest advancements in serum biomarkers for detecting hepatic fibrogenesis and advocates for concerted efforts to consolidate current knowledge, thereby providing patients with early, effective, and accessible diagnoses that facilitate personalized therapeutic strategies.

## 1. Introduction

Liver fibrosis is a chronic inflammatory condition affecting hepatic tissue, characterized by the progressive replacement of normal parenchymal cells with excessive extracellular matrix (ECM) components [1]. This dynamic process is necessary to limit liver injury triggered by inflammation [2]. However, the resulting condition leads to the establishment of several mechanisms that significantly alter both the histological and the macroscopic architecture of the liver [3,4,5].

The progression of the disease generally spans 15–20 years (but it must be noticed that the leading-cause disease should be considered as it can influence the timing of disease progression), advancing to severe stages known as cirrhosis, as well as hepatocellular carcinoma (HCC) and liver failure [6,7,8].

Chronic liver diseases (CLDs) are among the major public health burdens worldwide, affecting approximately 800 million people, with an average mortality rate of about 2 million deaths per year [9,10,11].

The incidence and prevalence of CLDs display a varied distribution across different geographic areas and are influenced by gender, race, and socioeconomic status. Furthermore, the etiology leading to CLDs introduces another layer of complexity to these conditions. Additionally, a significant percentage of patients remain asymptomatic until they require liver transplantation, or in the worst cases, are only diagnosed pre-mortem [9].

Animal models and clinical studies have shown that the progression of liver fibrosis can be halted or reversed if the causative factor is eliminated. However, the “point of no return” is sometimes indeterminate, and simply removing the cause may not always be effective [12,13].

In recent years, hepatology research has concentrated on developing straightforward and accurate methods for both diagnosing and stratifying the disease, which could be utilized during therapy and follow-up. This review aims to summarize the current state of knowledge on liver fibrosis, focusing on the causes of liver damage, the tissue’s response, and the latest methods for early diagnosis that lead to effective therapy.

## 2. Main Associated Etiologies

Chronic liver diseases can be described as dynamic clinical pictures, exhibiting various manifestations depending on the etiology of the disease. These can be categorized into five main clinical scenarios: (I) chronic infection by hepatotropic viruses such as HBV—hepatitis B virus—and HCV—hepatitis C virus [14]; (II) alcoholic liver disease (ALD) [15]; (III) Non-Alcoholic Fatty Liver Disease (NAFLD), lately named as metabolic dysfunction-associated steatotic liver disease (MASLD) [16,17]; (IV) autoimmune liver diseases such as primary biliary cirrhosis (PBC), primary sclerosing cholangitis (PSC), and autoimmune hepatitis (AIH) [18,19]; (V) hereditary diseases, such as Wilson’s disease, hemochromatosis, and alpha-1-antitrypsin deficiency [20].

Having made this distinction, it becomes clear that different populations (according to gender, age, geographical locations, social status, and general lifestyle) are susceptible to diverse factors that contribute to the disease. Moreover, understanding the specific etiology is crucial. It significantly influences the progression of the disease through various morphological patterns of fibrosis, depending on the cell types and mechanisms active within the liver microenvironment [13,14,15,16,18,19,20]. These factors give rise to multiple forms of liver fibrosis, where the morphological changes and the timing of progression display unique characteristics, which need to be considered in the development of novel diagnostic methods and subsequent therapeutic strategies [21].

## 3. Main Aspect of the Pathophysiology of Liver Fibrosis

Liver fibrogenesis is a dynamic condition in which a meshwork of molecular, cellular, and tissue processes work closely together, leading to the progressive accumulation of ECM components. This process has two main hallmarks: an increased deposition that affects both the quality and the topographic distribution of ECM components, and a change in the remodeling of the ECM, caused by the altered expression of genes coding for matrix metalloproteinases (MMPs) or tissue inhibitors of metalloproteinases (TIMPs) [22,23,24,25].

The primary cell type involved in the development of liver fibrosis is the hepatic myofibroblast (MF). This is a heterogeneous population with a strong proliferative rate and profibrogenic abilities [26,27]. Hepatic myofibroblasts can originate from different mesenchymal precursor cells through activation/trans-differentiation processes [20,28]. However, the major source of MFs is HSCs (hepatic stellate cells) [8,29,30], while the contribution from other sources remains controversial [20,31,32].

The role of MFs in liver fibrosis is crucial. They are responsible for the massive production and deposition of extracellular matrix (ECM) components. Additionally, they release endothelin-1, a potent vasoconstrictor that promotes proliferation, fibrogenesis, and contraction, which has been linked to portal hypertension. Moreover, HSCs/MFs release TGF-β1 (transforming growth factor-β1), which induces the production of fibrillary collagen type I and III, α-SMA (α-smooth muscle actin), laminin, and fibronectin, as well as VEGF-A (vascular endothelial growth factor), angiopoietin-1 and -2, PDGF-BB (platelet-derived growth factor-BB), contributing to their proangiogenic role [29]. They can modulate inflammatory and immune response, as well as angiogenesis processes. 

Overall, persistently activated MFs can integrate incoming paracrine/autocrine signals (reactive oxygen species (ROS), growth factors, cytokines, chemokines, adipokines, proangiogenic mediators, hormones and metabolism products) from the profibrogenic environment and released by both hepatic and extrahepatic population involved in CLD progression [33,34]. The result is the establishment of a feedback loop involving cross talk between different cellular populations, ultimately leading to a more severe stage of CLD [35,36].

Alongside this, another main process occurring during CLDs is angiogenesis. It consists in the formation of new blood vessels from pre-existing ones and can follow either a physiological or a pathological pattern. This process is involved in all fibrosis stages, from the earlier to the latest and is the major responsible for portal hypertension. 

The main driving forces of angiogenesis are hypoxia and hypoxia-inducible factors (HIFs), together with the main representative cell types involved in fibrosis, namely MFs and HSCs/MFs. These cells contribute to the inflammatory and pro-/anti-angiogenesis response, leading to a vicious circle where fibrosis causes inflammation and hypoxia, thereby increasing the need for new vessel formation. However, these new vessels are not mature enough to allow efficient perfusion for liver regeneration. Consequently, hypoxia is not corrected, and liver fibrosis and the disruption of normal tissue are promoted. Notably, this process is crucial for certain types of fibrosis, such as those caused by chronic viral infections and autoimmune diseases, characterized by bridging and postnecrotic fibrosis. In these cases, angiogenesis is more produced than in other fibrosis etiologies (NAFLD or MASLD, ALD or biliary fibrosis). Moreover, the resolution of fibrosis in these cases has a worse prognosis [3,37].

The main pathway that carries on this process seems to be the angiopoietin/Tie-2 pathway. It involves the ligands angiopoietin-1 and -2 (Ang-1 and Ang-2), which bind to their common tyrosine kinase receptor Tie-2. Ang-1 supports autophosphorylation, while Ang-2 suppresses phosphorylation. This interaction defines Tie-2 activity related to vascular stabilization and remodeling, or vascular regression in cooperation with VEGF [38].

The importance of angiogenesis in CLDs relies on the fact that anti-angiogenic therapies have shown good responses in both experimental models and patient cohorts [3,39].

Considering the immune cells involved in liver fibrosis, the first committed are Kupffer cells (KC) and monocyte-derived macrophages (MoMF). When liver injury occurs, depending on their polarization, these cells can either promote the restoration of tissue integrity in the case of acute injury or contribute to the progression of the disease in chronic cases [40]. The activation of KCs after damage-associated molecular pattern (DAMP) and pathogen-associated molecular pattern (PAMP) binding leads to the release of proinflammatory cytokines and chemokines [41,42,43,44]. This activation is followed by the recruitment of circulating leukocytes (monocytes and neutrophils), modulation of T lymphocytes, and increased expression of vascular adhesion molecules on sinusoidal endothelial cells (SEC) [45]. Among other immune cells, neutrophils apparently do not sustain fibrogenesis but rather contribute to collagen degradation during the resolution of injury by releasing MMPs. In contrast, dendritic cells (DC) seem to have a profibrogenic role [46]. Cells derived from naive CD4+ (cluster of differentiation 4+) lymphocytes can play a role in modulating fibrosis. TH17 lymphocytes (thymus-derived lymphocytes helper 17), which release proinflammatory interleukin-17A (IL-17A), appear to have a profibrogenic role [47,48]. Conversely, TH1 lymphocyte responses, as well as NK (natural killer) or NK-T cells, are usually anti-fibrotic, mainly through interferon-γ (IFN-γ) mechanisms. The role of T regulatory cells in chronic liver diseases (CLDs) is more complex, while limited evidence is known about B lymphocytes [49].

**Figure 1:**
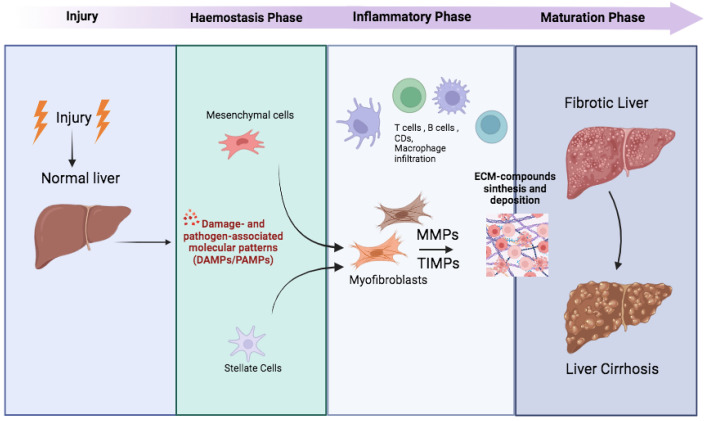
Cascade of fibrotic process activation in liver diseases. Physiologically, there are four distinct phases, injury, hemostasis, inflammatory phase, and maturation phase, which lead to the regeneration of damaged tissues following an injury. In the liver, when the wound response becomes pathogenic, the generation of fibrotic tissue replaces liver tissue and impairs organ function. The onset of fibrosis occurs with the activation of quiescent HSCs, i.e., resident mesenchymal cells. These cells differentiate into myofibroblasts and begin to secrete ECM constituents, particularly increasing the expression of fibrotic collagens (i.e., types III, IV, and V), fibronectins, and hyaluronic acid. Other pro-fibrotic components have also been implicated as endogenous DAMPs recognized by PRRs (pattern recognition receptors). MMPs, expressed by a variety of immune and non-immune cells, degrade ECM components, including collagen and fibronectin, making them essential for tissue remodeling. The balance between MMPs and TIMPs in the liver plays a vital role in the induction of liver fibrosis.

This complex scenario of cellular and molecular interactions must be analyzed individually for each patient affected by CLDs, as different etiologies lead to the development of unique pathways and cellular strategies [42] (Figure 1).

## 4. Main Diagnosis Strategies 

### 4.1. Liver Biopsies

Liver fibrosis affects both the morphological and histological structure of the liver. Therefore, clinical evaluation has relied on the histopathological assessment of liver tissue fibrosis obtained from liver biopsies. In the 1980s, semi-quantitative scoring systems were introduced. These systems allowed for the definition of grading and staging based on liver biopsies.

The aim was to standardize interpretation for both pathologists and clinicians.

Liver biopsy results rely on the EASL-ALEH Clinical Practice Guidelines and METAVIR classification [40,50,51]. Here, (fibrosis stage) F0 (no fibrosis) is a state with no detectable signs of fibrosis. In F1 (mild state), fibrosis is limited to the portal zone and only minimal signs of scarring are visible. Significant fibrosis is characterized by a METAVIR score of F2 (moderate) and an Ishak score ≥3 or greater. In this state, there is involvement of the portal zones and the formation of occasional bridging and septa, with scars around vessels within the liver. Later, F3 (severe state) features marked bridging and occasional nodules. A score such as METAVIR F4 with an Ishak score ≥5 indicates a state of cirrhosis [52,53,54].

**Figure 2:**
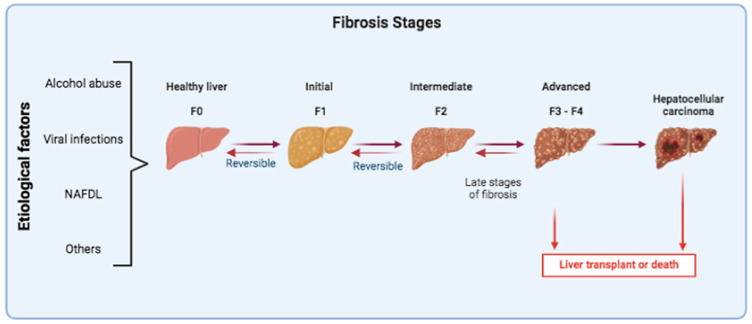
Liver fibrosis stages. Persistent liver damage, regardless of its various etiologies, leads to a progressive deposition of fibrous tissue and alteration of the normal liver parenchyma. The METAVIR fibrosis score classifies fibrosis into five possible stages: F0 = no fibrosis, F1 = portal fibrosis without septa, F2 = portal fibrosis with rare septa, F3 = numerous septa without cirrhosis, and F4 = cirrhosis. Ultimately, cirrhosis can progress to liver cancer. The progression of liver fibrosis can be interrupted or reversed with the elimination of the hit in the early stages F1 and F2, while the advanced stages are not easily reversible, in fact only proper therapies could revert the stage of disease. The only alternative for the patient’s recovery is a liver transplant.

Liver biopsy remains the gold standard for CLD diagnosis today, but it has inherent characteristics that can induce mistakes. It is a procedure in which those who perform the biopsy procedure and those who read the final product can introduce crucial errors in the diagnosis. Another drawback is that diagnosis is made on a biopsy specimen that reflects only 1/50,000 of the liver. Considering the heterogeneous distribution of fibrosis within the liver parenchyma, results can be misleading [55]. Lastly, it is an invasive and costly surgical procedure. Patients are exposed to risks of pain, bleeding, and major infections [56]. Overall, problems concerning interpretation, reproducibility, and sampling errors in biopsies remain unresolved. Thus, a re-evaluation of the role of liver biopsy in CLD diagnosis is needed (Figure 2).

### 4.2. Imaging Techniques 

Standardized morphometric analysis of liver tissue fibrosis, known as computer-assisted morphometry, could provide a quantitative measure of hepatic fibrosis and could reduce intra- and inter-observer variability when standardized. Morphometric analysis using the Collagen Proportionate Area (CPA) system has demonstrated a positive correlation between the amount of fibrosis in cirrhotic liver and the relative hepatic vein pressure gradient (HVPG) and liver tissue stiffness. Other non-invasive alternatives based on imaging techniques are available such as ultrasound (US) [57], magnetic resonance imaging (MRI), and FibroScan transient elastography [58,59]. These are quick and safe methods widely used to assess fibrosis and cirrhosis. Conventional magnetic resonance imaging (MRI) provides information about macro-structural and parenchymal changes characteristic of liver fibrosis and cirrhosis, which can be improved by the administration of intravenous contrast material. 

Elastography techniques are a group of methods known for their accuracy as they directly measure liver elasticity and stiffness. Among these, we should mention Vibration-Controlled Transient Elastography (VCTE)_or FibroScan and Magnetic Resonance Elastography (MRE). Each technology differs from the others in the quality of the impulse used for evaluating the disease. Therefore, different measures provided by different techniques yield results that cannot be compared [60].

As FibroScan is the recommended method to assess the presence of fibrosis, it should be noted that patients with ascites and/or obesity may obtain unreliable results or experience scan failure; a factor which can also be influenced by operator expertise [60].

MRE is a specialized MR technique with very high accuracy (AUC = 0.90) and a low technical failure rate (≤5%). While it may perform better than VCTE, its effectiveness is similarly reduced in patients with ascites and obesity [60].

Its drawbacks include high costs (as a matter of fact, it is available only in specialized clinical settings) and potentially unreliable results in patients with ascites and/or obesity. This last one, is an important point against this method, as most patients belong to these categories [60,61].

### 4.3. Non-Invasive Tests and Tools

The introduction of quantifiable laboratory tests and their correlation with physiological and pathological conditions has been very useful for clinicians and researchers. In fact, non-invasive measures have been increasingly introduced, changing the clinical management of CLDs over the last 15–20 years [62,63]. These measures are based on non-invasive, cost-effective, repeatable, safer, and better tolerated markers, which lead to (I) the earlier detection of hepatic fibrosis in patients and (II) new stratification and prognostication models. Available non-invasive tools overcome the limitations of biopsies and aim to identify significant fibrosis and cirrhosis. They rely on simple scores calculated from routine laboratory parameters or more complex serum biomarkers that consider circulating components, derived from the accumulation or the remodeling of the ECM, and are associated with elastography techniques to measure liver stiffness. Here, we can split them into: (I) class I or direct biomarkers, those that reflect changes in ECM structure (turnover, fibrogenesis, fibrolysis); (II) class II or indirect biomarkers, those related to liver damage and the decline of liver function due to fibrosis or cirrhosis [64].

The endpoints under trial are dependent on the histological scoring system mentioned above. Although the accuracy of non-invasive tests or tools remains controversial, early evidence suggests they could be as effective as liver biopsy [62].

The first quantifiable non-invasive markers were serum levels of liver enzymes such as alkaline phosphatase and transaminases (alanine aminotransferase—ALT, aspartate aminotransferase—AST) known as liver damage tests, as they are released from damaged cells. Transaminases are mainly present in the hepatic and muscular cells, particularly in the cytoplasm (ALT and AST) and mitochondria (AST). Their cutoff is established at up to 60 U/L for men and 42 U/L for woman for ALT and up to 35 U/L for AST. An increase in transaminases concentration is synonymous with liver necrosis. Otherwise, they exhibit changes during the day, increase with the body mass index and after bodily activity. Hemolysis and the conservation of samples can also influence the results. The clinical significance of these tests is enhanced by the AST/ALT ratio. Generally, the first markers that can be identified in liver injury are the increase in transaminases and bilirubin, as the latter is indicated as a marker of severe liver tissue and function damage [63].

Thereafter, these were correlated with the platelet count. Fibrosis and cirrhosis can be readily detected by the APRI test (AST to Platelet Ratio Index). In fact, thrombocytopenia is a common complication of CLDs due to splenic platelets’ sequestration, immune-mediated peripheral destruction, bone marrow suppression caused by HCV infection and its treatment, or by a decreased concentration of hematopoietic growth-factor thrombopoietin [65,66]. This test was evaluated in HCV and HBV patients. With an assumed cutoff value of 0.5, the APRI test showed 81% sensitivity and 50% specificity for detecting significant fibrosis, with an NPV of 80%. An APRI value >1 in the general population indicates a high risk for advanced fibrosis [60].

Many other serum enzymes easily tested in the laboratory could be considered:

- Bilirubin.
- α1-fetoprotein (AFP), an oncofetal protein, used as marker for HCC [67].
- α-2-macroglobulin (A2M), a proteinase inhibitor synthesized by hepatocytes. Its concentration is age-dependent [68].
- Haptoglobin (Hp) is a glycoprotein synthesized in the liver and in the lung. It plays different roles in tissue protection, the prevention of oxidative damage, and regulatory function. It is known that its concentration rises during acute-phase inflammation but also in obese patients [69,70].
- Apolipoprotein-1 (ApoA1) is a high-density lipoprotein that functions as anti-atherogenic agent with defined role in liver steatosis and cirrhosis [71].
- Albumin.
- Gamma-glutamyl transpeptidase (GGT) is an enzyme present on the cell membrane of various cells, with a major contribution to the serum concentration in the liver. This varies by sex, age, and ethnicity. Moreover, different stages of increase are predictors of different diseases.
- Ferritin is a biomarker for total body iron stores. The increase in its concentration corresponds to an acute-phase inflammation reaction, increased production, or increased leakage from damaged hepatocytes. Ferritin’s concentration varies by sex and age [72,73,74].

Moreover, the role of the liver in glucose metabolism is crucial as the liver is the major contributor to endogenous glucose production and the largest reserve of glycogen. Glucose metabolism is included in different tools as it is known that alteration of the liver reflects in glucose metabolism dysfunction and metabolic syndrome [75,76].

Although all of these are good markers, they underperformed in disease specificity and show differences from physiological concentration in other inflammatory diseases as well [62,63,77].

The next step was to test the effectiveness of a combination of markers, considered not relevant as a single measurement (without a clear-cut prognostic use). In general, they exclude advanced fibrosis and cirrhosis but are not able to discriminate between early and mild fibrosis [78,79].

In Table 1, we summarize some of them. 

The rise in the ALT/AST ratio is closely associated with the presence of cirrhosis in patients with chronic viral hepatitis and NAFLD (or MASLD). The APRI can diagnose fibrosis and cirrhosis with acceptable accuracy. It allows for the exclusion of advanced but not moderate fibrosis and facilitates the management of the follow-up in HCV patients treated with telaprevir. It is recommended alongside ultrasound scanning for the diagnosis of NAFLD (or MASLD) and NASH. The performance values of FIB4 and FibroTest are comparable [80]. Their scores correlate with liver damage, and their validity for diagnosing advanced fibrosis and cirrhosis has been confirmed. Scores can be influenced by both treatment and inflammation. FibroTest is patented by Biopredictive. Fibrometer and Cirrhometer are patented by Echosens. The coefficients included in the Cirrhometer are specific to cirrhosis. FIB4, APRI, and Fibrometer were better than a METAVIR fibrosis score at the baseline at predicting serious liver-related events [80]. The Cirrhometer was the only tool that predicted liver-related death. The combination of Citometer and Cirrhometer yields the best non-invasive score among those mentioned, although further evaluation is required. The ELF panel is an algorithm developed by the European Liver Fibrosis Group based on the analysis of HA, TIMP-1, and PIIIPN. Its performance should be considered for identifying advanced fibrosis in NAFLD/MASLD, AC (alcoholic cirrhosis), and methotrexate-induced liver fibrosis, although it is influenced by age and gender, particularly in HCV and HBV patients. There are three formulas available for this algorithm (developed by Guha and Siemens), and its uses is recommended by the National Institute for Health and Care Excellence for the management of NAFLD. The ELF test initially included age as one of its variables, but it was shown that omitting age did not alter the results. In contrast, Hepascore and Fibrotest/FibroSure include sex and age [58]. MAF-5 is a straightforward tool (with a freely available formula) that correlates weight status (body mass index (BMI) and waist circumference (WC)) with lipid and glucose metabolic status, transaminases, and platelets. The result is a risk score that should be used as a screening tool in at-risk population to identify at-risk individuals, independently of age [83].

The unsatisfactory results have prompted efforts to develop new markers related to the pathological process in order to use them in an integrative manner with the aforementioned molecules. 

Direct markers, which include products of the ECM metabolism, are currently considered experimental and have not been widely accepted in clinical practice. As liver fibrosis is associated with an alteration of the composition of the ECM, all its components (HA, PIIIPN, type 4 collagen, laminin, microfibrillar-associated protein 4—MFAP4) are under study [63,81,84,85,86].

Here, we can mention hyaluronan or hyaluronic acid (HA), which is found in the ECM. Its concentration reflects its turnover. Physiologically, it is rapidly degraded by hepatic endothelial cells, and its half-life in blood is about 2–5 min. High levels of HA in serum reflect increased production or reduced degradation, thus inflammation and fibrosis. The upper limit of the normal range is defined as 98 µg/L [87,88]. Furthermore, its concentration directly correlates with the stage of fibrosis in HBV and HCV patients [54,62,63,89,90,91].

The second mentionable marker is the N-terminal pro-peptide of collagen type III (PIIINP) the precursor of collagen synthesis by activated HSCs. Although initially, it demonstrated 94% sensitivity and 81% specificity in detecting cirrhosis and correlation with aminotransferase levels, it is a better marker of inflammation rather than fibrosis. Its concentration increases in AC and cholestasis. It also permits clinicians to differentiate patients with chronic HBV. Otherwise PIIINP levels cannot help in stratifying between mild, moderate, and severe fibrosis [54,63,92,93].

Type IV collagen level was also investigated, with a better correlation with fibrosis. Its concentration rises in HBV, HCV, and NASH patients proportionally to the fibrosis state [63,94].

Glycoprotein YKL-40 is expressed in various tissues including liver, particularly in HSCs. It is involved in inflammatory processes such as chemotaxis, cell attachment and migration, reorganization, and ECM remodeling in response to endothelial damage. Its serum concentration is higher in inflammatory disease, including liver fibrosis [95,96]. Its trend is the same as that of the other direct markers mentioned above [63,77,92].

Laminin levels have been evaluated for fibrosis in HBV patients. It could also be used in the assessment of the stage of liver fibrosis in HCV patients during the follow-up phase of drug treatment for both conditions [54,63,97].

The last studied molecule is cholylglycine—CG. It is the combination of bile acid and glycine. CG has been shown to be useful in liver disease diagnosis and prognosis as it reflects the damage degree of liver cells. Under physiological conditions, its concentrations are lower than those in liver damage and increase in hepatobiliary diseases, viral hepatitis, ALD, cirrhosis, and HCC [62,98,99,100].

Cytokines have been investigated as biomarkers of fibrosis too. Among them, we can mention TGF-β, whose levels correlate with the presence of fibrosis either in ALD or in HCV patients and TNF-α, which correlates with fibrosis in ALD and HBV patients. [63,101]. Connective tissue growth factor (CTGF) is closely associated with fibrosis status, and its levels decrease in cirrhosis [77]. 

Here, the importance of looking at transforming growth factor β1 (TGF-β1) is because it is activated from deposits in the ECM or released by various cell types during liver injuries. Moreover, it is defined as the most representative among the pro-fibrotic cytokines involved in the initiation and progression of fibrosis throughout a peculiar mechanism that includes the transformation of HSC/MFs. The cutoff to determine the stability of the disease is 75 ng/mL, but it suffers from platelet contamination [63,102,103,104]. Transforming growth factor α (TGF-α) enhances the proliferation of HSCs, and it has been shown that its concentration changes with disease progression [63]. Connective tissue growth factor (CTGF) is a small protein involved in the regulation of the ECM’s synthesis and production. Its serum concentration is increased in patients with liver fibrosis. Although it is known for its role in cell adhesion, migration, angiogenesis, MF activation, and ECM deposition and remodeling, its mechanism is not deeply known, so it is interesting for research [105,106].

Among cytokines, a noteworthy one is osteopontin (OPN) or secreted phosphoprotein 1. It is a ubiquitous cell-signaling protein involved in physiological and pathological events. OPN can be attributed to liver, kidneys, and bones and is correlated with pathological conditions such as inflammation, angiogenesis, fibrosis, and carcinogenesis. In liver, it is secreted by hepatocytes in pathological conditions and serves as a cytokine in the ECM signaling, promoting fibrogenesis through a mechanism not clearly understood. Further connections with the aforementioned causes of fibrosis and chronic liver diseases include OPN’s ability to promote HSC activation, proliferation, and migration. Additionally, its signaling is enhanced through its degradation products, and its interaction with the immune system plays a role in promoting CLDs. OPN has been found to be upregulated in NASH and NAFLD/MASLD patients. There, the OPN expression correlates with steatosis and insulin resistance in obese patients, and serum OPN concentration correlates with liver fibrosis. Thus, it was suggested as a NIT (non-invasive test) for liver fibrosis in NAFLD/MASDL patients [107]. Its correlation with liver fibrosis is also positive in HCV and HBV patients, who show higher OPN plasma concentration, according to the fibrosis stage [108]. OPN plasma concentration could be useful in the assessment and stratification of liver fibrosis; furthermore, it can be an “alarm” for critical patients at risk of portal hypertension, as its concentration correlates with HVPG (hepatic venous pressure gradients) [109]. OPN values are significantly increased in HCC patients and have showed positive correlation with AFP. A higher concentration of OPN corresponds to major OPN gene expression and correlates with a histopathological analysis [110,111,112,113].

In addition, interleukins (IL) have been included in various studies investigating new effective biomarkers. Specifically:

- IL-33, a member of the IL-1 family, plays a role in mast cell activation, Th2 differentiation, and dendritic cell development in bone marrow cultures. Furthermore, it has a clear proinflammatory role in autoimmune diseases, allergic diseases, and chronic inflammatory diseases. Its role extends to the host response to viral infections such as HIV, HCV, and DENV (dengue virus). Increased levels of IL-33 are related to liver damage in CHC patients and to the development of HCV/HBV infection into liver fibrosis. IL-33 serum levels are significantly elevated in CHC and HCC patients compared to healthy controls, but no significant difference was observed between them. In particular, serum levels of IL-33 are significantly correlated with the HCV RNA load and are higher in the latest stages of fibrosis than in the earlier stages, according to METAVIR scores. IL-33 levels are higher in fibrosis and seem to positively correlate with the development and progression of fibrosis and liver damage. IL-33 serum levels correlate significantly with TGF-β1 serum levels. In particular, IL-33 promotes the activation of macrophages, which promote TGF-β, further upregulating IL-33 expression. This correlation strengthens their role in the fibrosis process. Moreover, IL-33 promotes the production of INF-gamma by NK cells. The latter, together with IL-6, plays an important role in liver injury and HCV infection. Their serum levels are higher in CHC patients and might indicate active viral replication and pathogenic progression. IL-33 can promote the production of IL-10 in macrophage-derived foam cells; thus, the levels of these two interleukins are correlated [114].
- IL-17 is a family of six members, one of which is IL-17E, also known as IL-25. They are mainly secreted by Th17 cells. IL-17 has a protective role against infections caused by extracellular pathogens as well as in chronic inflammation, autoimmune diseases, and tumor growth. Additionally, it plays a role in the adaptive immune response against HCV and HBV. IL-17 is frequently elevated in patients with liver cirrhosis, autoimmune hepatitis, steatohepatitis, and alcohol-related HCC. In fact, it is associated with liver inflammation and damage, contributing to disease progression. IL-17 serum levels are significantly higher in CHC and HCC patients compared to healthy controls; furthermore, HCC patients show higher levels than CHC patients. These levels correlate with the degree of liver fibrosis, even though they do not correlate with HCV RNA loads. Serum IL-17 levels are higher and correlate with ALT levels in HBV patients. Given that IL-17 concentrations are linked to HCC, some groups have studied its relationship with AFP levels and how these predict HCC occurrence over a 4-year period. In this context, IL-17 concentrations appear to be useful for significantly identifying patients at risk during the next 4 years. Combining IL-17 with AFP measures with an available free formula result in a risk score with better performance than IL-17 and AFP alone [114,115].
- IL-25, also known as IL-17E, regulates Th2 responses against helminthic parasites and allergic inflammation. IL-25 serum levels are significantly higher in HCC patients compared to CHC patients and healthy controls [114].
- IL-10 is known for its dual role; in fact, it plays in both immune suppression and immune stimulation. It is associated with a worse prognosis in HCV patients. IL-10 serum levels are increased in patients with CLD, including hepatitis, cirrhosis, HBV, HCC, and CHC. These levels are closely associated with disease progression and inflammation and correlate with ALT serum levels. Higher levels of IL-10 are associated with a poor prognosis in several types of cancer. IL-10 has been assessed as a marker for HCC to predict postoperative recurrence, as its serum levels decrease after tumor removal. However, it has not been validated for clinical use. IL-10 could be used as a complementary tumor marker to the traditional AFP to identify a subset of HCC patients with a low AFP level. IL-10 levels may be related to hepatic injury caused by cirrhotic processes rather than tumor load. Additionally, IL-10 offers additional prognostic value to the existing tumor staging system [114,116,117,118].
- IL-6 is a pleiotropic cytokine with multiple physiological and pathological functions. In physiological conditions, its blood and interstitial concentrations are extremely low. These levels increase with aging, inflammation, and pathological conditions, particularly in liver disease. IL-6 is expressed by HSCs after HIF-1α induction. During liver inflammation, hepatic IL-6 levels can be more than 100 ng/mL. IL-6 induces acute-phase inflammatory proteins during infection. Chronic exposure to IL-6, due to chronic inflammatory insults, determines the setting up of chronic liver disease. Higher concentrations of IL-6 are found in several CLDs, such as NAFLD/MASLD, NASH, HCC, hepatocarcinogenesis, and the progression of the disease with a poor prognosis. IL-6 does not correlate with AFP. In addition, there is no significant survival difference between patients with high or low levels of serum IL-6 [117,118,119,120,121,122].

Other molecules studied are MMPs and TIMPs. Tissue Inhibitor of Metalloproteinases-1 (TIMP-1) is expressed after the interaction between hepatic myofibroblasts and liver macrophages. TIMP-1 is upregulated during hepatic fibrogenesis and promotes fibrosis in injured liver by inhibiting matrix metalloproteinases (MMP) and the degradation of the ECM. Thus, during fibrosis, the concentration of the former decreases while the latter increases. However, these have a better correlation with cirrhosis rather than fibrosis [25,81,123,124,125].

Another experimental marker is Cytokeratin-18 (CK18), a protein abundant in hepatocytes and cholangiocytes and used as marker of cell damage and death, which can predict severe fibrosis in alcoholics. Furthermore, its concentration has been studied in different situations including CLDs as marker of hepatocytes apoptosis (drug-induced liver injuries, HCV, NAFLD/MASLD) [126,127,128,129,130].

GP73, Release of Golgi protein-73, better correlates with cirrhosis in different CLDs. It is a transmembrane protein also found in the Golgi apparatus in biliary epithelial cells and hepatocytes, and it shows a peculiar expression in hepatocytes during acute and chronic liver disease [131,132,133,134].

Ten-eleven translocation protein (TET3) is an enzyme involved in DNA demethylation expressed in liver tissue. DNA methylation can have an important role in liver diseases. There, TET3 can activate genes through DNA demethylation, which may reflect in the development of a variety of liver diseases [135,136,137]. TET3 in fibrosis patients has been demonstrated to be higher than in control cases. In combination with FIB-4, it seems to be a promising non-invasive tool [77,135].

Ferritin together with BMI (body mass index) could be useful for individuating advanced fibrosis and cirrhosis in NAFLD/MASLD patients. Finally, the concentration of biomarkers of oxidative stress, such as malondialdehyde (MDA) and superoxide dismutase (SOD), correlates with fibrosis and cirrhosis in HCV patients [77]. In fact, reactive oxygen species and oxidative stress-related metabolites are under studies, as oxidative stress is considered one of the major factors leading to and promoting liver disease establishment and progression [138,139,140].

Overall, these markers show a correlation between their concentration and fibrosis or cirrhosis state, but they are not liver-specific, as they can be released from other damaged tissue [55].

Among cytokines mediators, Interferon Lambda 3 (IFN-L3), which increases in advanced fibrosis, can be more specific to the liver during HCV infection. Evidence suggests INF-L3 is a determinant of liver inflammation and fibrosis, whatever its etiologies [140].

Other approaches focus on the study of proteome during the development of CLDs. Some markers identified are microfibril-associated protein-4 (MFAP-4), the α2 macroglobulin (A2M)/hemopexin ratio, and vitamin D binding protein (VDBP) [77]. 

A genome-wide transcriptome study has outlined 122 HSC-specific genes and 194 HSC-specific gene signatures associated with a poor prognosis and an easier development of HCC in patients with CLD. Omics approaches to studying CLD in patients appear to be a promising strategy, but they require further development and represent a more complex option for clinical use [60,63,141,142,143].

Overall, the best experimental markers identified are the following: (I) serum transferrin, whose concentrations are higher in mild fibrosis (F1, F2) and decrease with the advancement of the disease (F3, F4); (II) serum levels of complement C3 and C4 beta chains that appear lower in HCV patients with cirrhosis; (III) asialoglycoprotein (sH2a), which is reduced in fibrosis and cirrhosis. Its prediction power increases in combination with ALT [77]. A soluble secreted variant of human asialoglycoprotein receptor (sH2a) is derived by the cleavage of the full receptor spliced variant in the endoplasmic reticulum of hepatocytes. While its plasma concentration is stable in healthy individuals, in liver disease, plasma concentration is reduced [144].

As mentioned in Section 3, angiogenesis is one of the main players responsible for liver fibrosis, particularly in chronic viral hepatitis. The leading pathway driving angiogenesis is the Ang/Tie-2 pathway. Several studies aim to correlate the serum concentrations of this pathway to identify new markers. Overall, Ang-2 serum levels correlate with the progression of the disease (fibrosis and angiogenesis and regression during treatment) in HCV patients and in those who develop HCC [39]. Higher levels of Ang-2 have also been observed in NAFLD/MASLD patients, which could be useful for distinguishing NASH from steatosis [145].

Meanwhile, Ang-2 serum levels increase with disease progression, Ang-1 levels decrease. The Ang-2/Ang-1 ratio also increases with the stage of fibrosis and shows better performance than the single measurement of Ang-1 and Ang-2. Exploring further, this measurement was linked to the traditional liver scores, resulting in a very promising composite index called AngioScore (AS) that combines Ang-2, age, INR, AST, platelet count, and GGT. This index, available through a freely accessible formula, has better performance than the other scores previously mentioned [37,146].

Another molecule that can identify whether vessels are new, or preexisting is PECAM-1 or CD31 (platelet endothelial cell adhesion molecule 1). It is a receptor expressed on the cell surface of several cell types: platelets, monocytes, neutrophils, lymphocytes, and endothelial cells. CD31 expression reflects the rate of endothelization of sinusoids and the extent of capillarization. The higher the expression, the worse/late the state of the disease, whereas the lower the expression, the more like normal the state under study is [147]. To strengthen the correlation between fibrosis and angiogenesis, a higher expression of CD31 is correlated with a higher expression of Ang1 and collagen-α1 in fibrotic liver [148].

All these non-invasive tests are better tolerated by patients as they are safer and easier to perform. Additionally, the cost is accessible for patients and laboratories. However, despite the promising aspects, they are not so good at detecting early fibrosis. Moreover, they are not liver-specific, as their concentration can be increased in different situations that are uncorrelated with liver fibrosis. It is important to underline that diagnoses cannot rely solely on serum results but must also involve imaging tests to address the lack of specificity.

Some tools combined biomarkers with the age or sex of the patient to increase the accuracy of the results [60]. It is important to have a large panel of tools available to obtain more accurate results. In particular, the more information included in the tools, the greater the accuracy of the results will be. 

To ensure the accuracy and reliability of the results, biologists and clinicians should pay attention to these points: (I) systemic inflammation may increase blood biomarker measures even if they are not correlated with liver fibrosis; (II) Gilbert or hemolysis and also the use of drugs or supplementation may increase biomarker measures (transaminases and bilirubin) independently from liver fibrosis status; (III) biomarkers have specific ranges of accuracy in correlation with age: NFS is less accurate in patients under 35 years old, and with the increase in age, its specificity decreases. Also, ALF increases with age [60]. FIB-4 lacks diagnostic performance in the under-40-year-old population [83].

Table 2 Summary of individual biomarkers under investigation for liver fibrosis diagnosis and stratification. Here we briefly introduce the link between serum/plasma markers mentioned and their role in liver disease.

### 4.4. Combined Non-Invasive Imaging and Serum Tests

To better identify patients at risk of advance fibrosis and cirrhosis within a selected cohort, some groups have developed formulas that combine characteristics of imaging and the previously mentioned tools, adding a layer of complexity and information. These are the FAST score and the Agile 3 and Agile 4 scores. The FAST score combines FibroScan and AST results to identify NASH patients at risk. This score has an AUC of 0.71, a positive predictive value of 33–85% and a negative predictive value of 73–100% [148]. The MAST Score, which combines MRI, PDFF (liver MRI proton density fat fraction), AST, and MRE + FIB-4 score, showed an improvement [149]. The Agile 3 and Agile 4 scores aim to assess the risk of advanced fibrosis and cirrhosis (stage 3 or 4) in NAFLD/MASLD patients by combining tools, biomarkers, and technologies currently used in clinical practice to enhance availability, experience, cost, and accuracy. These panels include liver stiffness measurement with VCTE (FibroScan), and serum biomarkers such as the AST/ALT ratio, platelet count, sex, diabetes status (Agile 4), and age (Agile 3). They represent models that predict a probability using those variables in a freely available formula on the Internet. The Agile 4 cutoffs are 0.251 and 0.565 with a sensitivity ≥ 85%, specificity ≥ 95%, and an AUROC of 0.90, with no more than 17% of indeterminate cases. The Agile 3 (which introduce the age variable) cutoffs are 0.451 and 0.679 with a sensitivity ≥ 85%, specificity ≥ 90%, and an AUROC of 0.90, with no more than 18% of indeterminate cases. Both are better than the results obtained using FIB-4 or FibroScan alone. However, further investigation is needed to clearly establish the cutoff, taking into account that the pitfalls rely on liver biopsies scores [148,150].

The use of the combination of these non-invasive tests allows clinicians to have a quick overview of the patient’s situation and determine if further investigation is needed to provide a prompt diagnosis and therapy. Defining the optimal range or cutoff values for disease remains challenging. Additionally, choosing the best score is a matter of debate.

The use of NFs, FIB-4, and APRI tests allows for the utilization of available and inexpensive laboratory tests with freely available formulas, enabling a quick determination of a patient’s risk for liver fibrosis. Thus, patients at low risk should repeat the test in 6 months or confirm the results with a combined biomarker panel or fibrosis imaging. High-risk patients should confirm the result with elastography and consider a liver biopsy or initiating treatment. Patients with indeterminate risk are typically first directed to other biomarker or imaging tests, such as ultrasound elastography (FibroScan), and ultimately, a biopsy if necessary [79,151,152] (Table 3).

**Figure 3:**
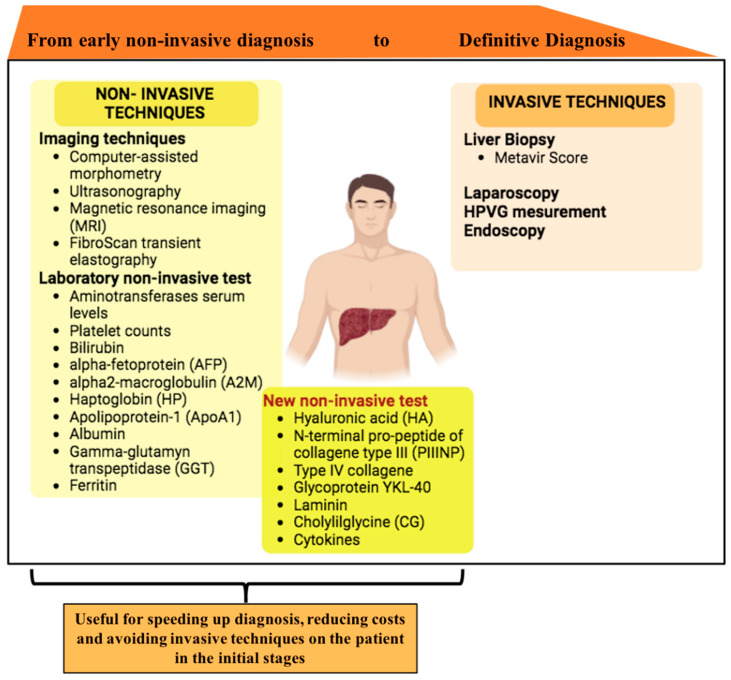
Summary of techniques used for staging liver fibrosis. In the left panel, non-invasive techniques such as imaging techniques and laboratory tests are mentioned. The new laboratory tests useful for staging are mentioned in the dark yellow box. Invasive techniques are listed in the right panel.

Despite the wide experimental literature that relies on non-invasive tests, none of them have undergone regulatory standard for approval (Figure 3). 

## 5. Discussion 

Currently, the gold standard for the diagnosis of liver disease is liver biopsy. However, due to its invasive nature, it is now considered a last resort in favor of promising non-invasive serum and imaging tests. The first liver biopsy was performed in 1883, and the scoring system developed in 1980 is still in use, allowing the standardization of pathological observations. Its sensitivity and specificity in detecting liver masses are 89.7% and 100%, respectively, with a diagnostic yield of about 87%.

However, liver biopsy has several drawbacks, as discussed in Section 4.1, primarily due to its invasive nature [153,154,155,156].

Imaging techniques developed since the second half of the last century, such as CT, show an AUC of 94% in predicting NASH but only 60% in predicting liver fibrosis. MRI techniques can classify 60% of cases unclassifiable by CT [157].

Overall, MRE is more accurate for liver fibrosis staging but is less accessible due to its high cost. In contrast, US elastography is valuable for its broad clinical use, greater availability, and cost-effectiveness [158].

The third group of strategies involves serum biomarkers. About 15% of patients with abnormal serum biomarkers who undergo liver biopsies show no abnormalities in liver histology [155]. This indicates that serum biomarkers alone can produce non-specific results. Combining markers or integrating serum biomarkers with imaging tests yields better diagnostic accuracy.

Overall, serum biomarkers are better tolerated by patients. As shown in Table 1, tools like the APRI test or FIB-4, involving measurements of AST, ALT, and platelet count, correlate well with liver biopsy results. This allows the stratification of the liver fibrosis risk in patients undergoing routine blood sampling. However, these measures are susceptible to various factors, which must be considered for an accurate liver disease diagnosis. For serum tests, commercial kits that provide multiple analytes in a single run can reduce costs and sample processing time. 

A probable difficulty is represented by differences among countries that have various healthcare systems and funding, affecting the availability of advanced diagnostic technologies and expert operators. Ensuring cost-effective and timely results in specialized facilities is crucial for efficient diagnoses.

In our opinion, first-step screening methods such as FIB-4, the APRI test, and US imaging are affordable and widely available, aiding in the stratification and identification of high-risk patients who should be referred to specialized facilities. 

Traditional markers like transaminases, platelet counts, bilirubin, and albumin are cheaper and useful for initial disease identification. In contrast, advanced biomarkers like TGF-B and PIIINP, while more expensive, offer a better stratification and follow-up potential, although their utility needs further validation. We believe that all tests and tools mentioned have potential if performed correctly and data are shared. Primary care physicians should be able to apply tests such as the AST/ALT ratio, APRI test, FIB-4, FibroTest, FORN index, Hepascore, Fibrometer, NFS, and MAF-5 score as needed, based on patient features. If liver disease is indicated, the second step should be US imaging, followed by FibroScan. Patients diagnosed with liver disease should then undergo further serum biomarker investigations (Table 2) to (I) correlate serum and imaging results; (II) monitor disease progression over time (e.g., IL-17 for HCC prognosis over four years); and (III) contribute to studies with small patient cohorts. Considering also the impact of lifestyle on liver diseases, awareness campaigns, screening processes, and the creation of networks between laboratories, clinicians, and pathologists are essential. They improve patient adherence, early identification, risk stratification, and timely introduction to therapy and follow-up processes. This approach will add valuable data for new screening, diagnosis, and stratification systems, potentially reversing disease progression and avoiding diagnoses when a poor prognosis precludes the chance of a cure.

## 6. Conclusions

Chronic liver diseases are a class of illnesses that progress through various stages of fibrosis, ultimately leading to cirrhosis, hepatocellular carcinoma, and liver failure [6,8,48,50,51,53]. These diseases, characterized by different etiologies, affect a significant portion of the world population and are expected to increase over the years [9,11,14,20].

Liver fibrosis is a chronic pathology that involves several cell types (MF, HSC, Kupffer cells, and MoMF), which form a tight network that may vary between etiologies [20,21].

The process could be halted or even reversed with the removal of the leading causative agent of the disease or through drug therapies [12,13,20]. Unfortunately, few pharmaceutical strategies are available, as diagnoses often come with a poor prognosis or the need for a transplant [12,13,20]. Furthermore, despite numerous research studies, only a few of the theoretical available approaches have been translated into clinical trials, and only a minority of these have received positive feedback from CLD patients [12,13,20,159,160]. Based on the above, hepatology is a field of interest in medical research today, and progress has been made. In particular, the scientific world has focused its attention on the evaluation of liver fibrosis staging as a prognostic value in the progression of specific CLDs and in the disease management, to prevent a poor prognosis such as liver cirrhosis, hepatocellular carcinoma, and/or the need for a liver transplant. 

The current method for detecting and determining fibrosis, as well as for classifying its stage, primarily relies on histological and morphological analysis, which has several drawbacks, including subjectivity, non-reproducibility, and a painful procedure [63,77,79].

The presence of various pathologies that contribute to the development of liver fibrosis, particularly the spread of metabolic conditions that lead to CLDs, necessitates new strategies that allow for an easy and timely assessment of liver fibrosis at a very early stage. 

To provide an early, easily accessible, reproducible, and rapid diagnosis, a re-evaluation of biopsy as the gold standard for the diagnosis of liver fibrosis is needed. However, numerous studies need to be conducted to determine physiological and pathological range values and stratify more useful biomarkers and tools based on specific etiology and specific cutoffs. Moreover, as for imaging techniques, different methods of analysis can influence the results [55].

Non-invasive tests, both serum and imaging-based, have provided an alternative to liver biopsy, offering an easier and more accessible diagnostic algorithm [79].

Among non-invasive tests, NF, FIB-4, APRI, and MAF-5 are cost-effective laboratory tests with readily available formulas. Based on these, clinicians can quickly determine the patient’s risk of liver fibrosis. Additionally, patients with undetermined risk should explore other biomarkers and/or imaging tests (such as Agile scores) or consider a liver biopsy. However, in experimental studies using non-invasive tests, there are no regulatory standards for approval [60,149,151].

Indeed, it is true that non-invasive serum-based tests are available and inexpensive, but they lack accuracy and specificity. Among these, better information is provided by class I or direct biomarkers, which often require dedicated expertise. More accurate information is provided by elastography methods that directly observe and quantify liver stiffness. Unfortunately, the reliability of the test is reduced in obese patients. MR elastography overcomes this problem but is expensive, and its availability in facilities is limited. Therefore, combining the availability, accuracy, sensitivity, and specificity of non-invasive serum tests and imaging tests may represent the best strategy to screen and confirm the presence of liver fibrosis in order to ensure early access to clinical protocols (therapy and follow-up). Here, the role of non-invasive tests during follow-up is limited because their accuracy does not seem to reflect the treatment-induced changes in fibrosis [60].

Moreover, defining a specific cutoff value for every biomarker or panel of them to determine the stage of liver fibrosis remains challenging. In this context, high cutoff values correspond to high specificity for advanced fibrosis and cirrhosis, while low cutoff values indicate greater sensitivity for the absence of fibrosis or early stages. Considering the low prevalence of advanced fibrosis or cirrhosis in the population tested with non-invasive tests, the positive predictive value of results near the high cutoff value is modest, and results should be considered alongside more clinical information. Meanwhile, the negative predictive value is more reliable for excluding advanced fibrosis or cirrhosis [60].

Liver fibrosis is a complex, inflammatory, and dynamic process influencing the histology and function of the liver. Its progression to advanced stages is a significant concern among affected patients who remain asymptomatic until a poor prognosis occurs. 

The stage progression of fibrosis is a significant cost in the management of patients who are asymptomatic until a poor prognosis occurs. 

The complexity of the disease depends on several factors: (I) different starting etiologies; (II) exposure of the population to genetic pathologies; (III) the presence of an unknown and feed-forward network between cellular and molecular signaling; (1) the accumulation of ECM components with increasing deposition and/or altered remodeling of the liver anatomy; (2) angiogenesis; and (3) the involvement of the immune system.

The staging of fibrosis relies on histological changes and is defined in the EASL-ALEH Clinical Practice Guidelines, METAVIR classification, and Ishak score, which are applied to liver biopsy specimens. The possible drawbacks of liver biopsies have shifted focus on new strategies for diagnosing and stratifying liver disease, such as imaging techniques (US, MRI–FibroScan, MRE) and serum biomarkers. The biomarkers are divided in direct biomarkers reflecting the liver ECM progression, and indirect biomarkers of liver function such as cytokines, liver metabolism, intracellular proteins triggering on the methylation of DNA, assessing or promoting angiogenesis. 

Prospectively, these non-invasive assays could evaluate different phases of disease progression but could be interesting for new biological therapies’ indication and patient selection alternatives to the liver biopsy considered the gold standard today.

In conclusion, non-invasive tests surpass liver biopsy in terms of patient compliance, reproducibility, accuracy, precision, and cost-effectiveness. However, they do not reflect the dynamic response to treatment or changes in fibrosis status, leaving room for promising developments in omics techniques, microbiome signatures, and the study of microRNAs, long non-coding RNA, and circular RNA [60,141].

Research should focus its efforts on providing a clear stratification of liver fibrosis for each different etiology to define specific cutoffs that reflect the FibroScan stratification of fibrosis, as it is the recommended method to evaluate the presence and stage of liver fibrosis, thanks to its ability to detect liver stiffness. In fact, different cutoffs can be the result of cohorts of patients with different etiologies that reflect a wider range of values. This would also provide the opportunity to define the most effective specific non-invasive score for each CLD-leading pathology [161].

In order to ensure early diagnosis, which gives more chances to access pharmaceutical protocols, optimal diagnostic strategies should integrate pathological events (fibrogenesis, angiogenesis, and liver regeneration), morphological imaging, and cellular products typical of disease progression. Further research based on the knowledge of the involved pathways can lead to new promising markers useful both in diagnosis and therapy.

Artificial intelligence could help clinicians and researchers find new targets and reduce the gap in ultrasound (US) and MRI techniques [162,163,164,165]. Finally, the study of non-invasive biomarkers represents an important field of research in recent years. Efforts should focus on determining the better (or the best) method and technology that permits available non-invasive and reproducible tests to establish some cutoff ranges that are currently unavailable. Considering the differences in liver disease resulting from various etiologies, further effort should be made to care for and distinguish between different patient populations (based on their etiology) and control populations [63,90,164].

## Figures and Tables

**Table 1:** Established serum marker tools.

Name	Biomarker Involved	Cutoff
Liver function test or liver damage test [77,80]	AST/ALT ratio	In chronic viral hepatitis, values of 1.0 are identified as the cutoff: below it, patients are not at risk of cirrhosis. Major values are seen in manifested cirrhosis.
APRI—AST Platelet Ratio Index [54,57,77,79,80,81]	AST (UI/L)/platelet count (10^9^/L) × 100	NAFLD/MASLD disease has a 1.0 cutoff: minor values identify low risk (NPV = 84%); major values identify high risk (PPV = 37%). AUROC: 0.67. In HCV infection, values below 1.0 identify low risk (NPV = 100%). Values greater than 2.0 identify high risk (PPV = 65%); intermediate values (1.0; 2.0) are indeterminate. AUROC: 0.94 In HBV infection, the cutoff is 1.0–1.5: less for low risk (NPV = 86%), more for high risk (PPV = 39%). AUROC: 0.75 It is utilized also in ALD, but cutoffs have not been identified yet.
FIB4—fibrosis 4 [54,59,63,77,79,80,81]	[age (years) × AST(UI/L)]/platelet count (10^9^/L) × ALT (UI/L)]^2^	In NAFLD/MASLD disease, values below 1.30 stand for low risk (NPV = 90%); values greater than 2.67 for high risk (PPV = 80%); intermediate values (1.30; 3.35) are indeterminate. AUROC: 0.80 In HCV infection, values lower than 1.45 identify a low risk for advanced fibrosis (NPV = 90%), values greater than 3.25 identify a high risk for advanced fibrosis (PPV 65%), and values greater than 5.88 stand for a high risk for advanced fibrosis (PPV = 82.5%). Intermediate values (1.45; 3.25) are indeterminate. AUROC: 0.765–0.83. In HBV infection, values less than 1.58 identify a low risk for advanced fibrosis (NPV = 84.6%) and values greater than 5.17 identify a high risk for advanced fibrosis (PPV 83.3%). Intermediate values (1.58–5.17) are undetermined. AUROC: 0.845
Fibrotest or FibroSure [77,79]	α2-Macroglobulin, apolipoprotein A1, haptoglobin, γ-glutamyl transpeptidase, bilirubin	In NAFLD/MASLD disease, the identified cutoff value is 0.30 (NPV = 97%). AUROC: 0.88 In HCV infection, the identified cutoff value is 0.52 (NPV = 94%). AUROC: 0.84 In HBV infection, the identified cutoff value is 0.48 (NPV = 90%) AUROC: 0.82
FORN index [59,63,79]	Platelet count, cholesterol levels, age, γ-glutamyl transpeptidase	AUROC: 0.76 for fibrosis detection and AUROC: 0.87 for cirrhosis detection
Hepascore [63,77,79]	HA, bilirubin, GGT, α2-macroglobulin, age, gender	In NAFLD/MASLD disease, the identified cutoff value is 0.37 (NPV = 97%). AUROC: 0.81 In HCV disease, the identified cutoff value is 0.47 (NPV = 95%). AUROC: 0.86 In HBV infection, the identified cutoff value is 0.42 (NPV = 90%). AUROC: 0.82
Fibrometer [63,79]	Glucose, AST, ferritin, platelet count, ALT, body weight, age	AUROC: 0.82 for fibrosis detection and AUROC: 0.91 for cirrhosis
Cirrhometer [79]	Fibrometer with specific coefficients	
NFS—NAFLD Fibrosis Score [77]	Age, body mass index, hyperglycemia, platelet count, albumin, AST/ALT Ratio	In NAFLD/MASLD disease, values <−1.455 stands for no advanced fibrosis/low risk (NPV = 88%), while values >−0.675 stand for advanced fibrosis/high risk (PPV = 82%). Intermediate values (>−1.455 and <−0.675) are indetermined. AUROC: 0.82–0.85
ELF panel—Enhanced Liver Fibrosis panel [63,77,81,82]	HA, TIMP-1, PIIINP	In NAFLD/MASLD disease, the identified cutoff is 10.35 (NPV = 94%). AUROC: 0.94 In HCV infection, the identified cutoff is 0.063 (NPV = 95%). AUROC: 0.773 In HBV infection, the identified cutoff is 8.4 (NPV = 88%). AUROC: 0.69
MAF-5 Metabolic Dysfunction Associated Fibrosis Score [83]	Waist circumference, body mass, AST, platelet count	In metabolic dysfunction, values less than 0 identify no fibrosis risk (NPV = 96.7%), while values greater than 1 identify a high fibrosis risk (PPV = 28%).

Table 1: Low risk for significant fibrosis stands for Kleiner or METAVIR score F0–2; advanced fibrosis F3–4; cirrhosis F4. NPV—negative predictive value; PPV—positive predictive value; AUROC—area under the receiver operating characteristic curve.

**Table 2:** Novel biomarkers of liver fibrosis.

Name	Analytical and Clinical Facts
Hyaluronic acid (HA) [54,62,63,89,90,91,97]	In HCV and HBV infection, the upper cutoff is 98 µg/L. AUROC = 0.79 for cirrhosis and 0.72 for fibrosis detection. It was found to have sensitivity and specificity ≥ 90% in detecting liver fibrosis, with an estimated accuracy of 86%.
N-terminal pro-peptide of collagen type III (PIIINP) [54,63,92,93]	In AC and cholestasis diseases and chronic HBV infection, it has 94% sensitivity and 81% specificity in detecting cirrhosis. For liver fibrosis detection, it was found to have a sensitivity and specificity ≥ 90%, with an estimated diagnostic accuracy of 74%.
Type 4 collagen [63,94]	Higher concentration has been found in HBV, HCV infection, and NASH patients.
Glycoprotein YKL-40 [63,77,92]	In HCV infection, it has an AUROC of 0.81 for fibrosis detection.
Laminin [54,63,97]	In HBV infection, it shows 71.9% sensitivity and 80% specificity for the assessment of liver fibrosis and during the follow-up. In HCV patients, it is used for the assessment of the liver fibrosis stage and during the follow-up. Estimated diagnostic accuracy of 81%.
Cholylglycine [99,100]	Its concentration increases in acute hepatitis/cirrhosis/liver damage.
TGF-β [63,101]	Its concentration correlates with disease progression. If it is less than 75 ng/mL, the disease is considered stable. There is also a correlation with ALD and HCV infection. The AUROC is 0.835 for the assessment of fibrosis. The estimated diagnostic accuracy is 67%.
TGF-α [63,101]	It is used in ALD and HBV infection.
Connective tissue growth factor (CTGF) [77]	Its values have shown an AUROC of 0.887 in correlation with fibrosis and an AUROC of 0.955 in correlation with cirrhosis.
Osteopontin (OPN) [107,108,109,110,111,112,113]	The AUC, sensitivity, and specificity in predicting any stage of fibrosis were 99%, 96%, and 100% in HBV patients and 97.4%, 96.5%, and 100% in HCV patients. Values over 80 ng/mL correlate with a high risk of portal hypertension, with 75% sensitivity and 63% specificity.
IL-33 [114]	Higher levels are detected in CHC and HCC patients compared to healthy individuals, according to liver fibrosis and HCV RNA loads.
IL-17 [114,115]	Higher levels are detected in HCC (greater levels) and CHC than in healthy patients, according to the fibrosis degree.
IL-17 + AFP [114,115]	Above the optimum cutoff of 4.5072, patients are at an increased risk of developing HCC, with a sensitivity of 100% and specificity of 79.9% (AUC = 0.933).
IL-25 [114]	Higher levels are detected in HCC (greater levels) and CHC than in healthy patients.
IL-10 [114,116,117,118]	IL-10 serum levels are increased in patients with several CLDs. These levels are closely associated with disease progression and inflammation. Higher levels are associated with a poor prognosis.
IL-6 [117,118,119,120,121,122]	Very low concentrations are detected under physiological conditions, but these concentrations rise to more than 100 ng/mL in CLD, such as NAFLD/MASLD, NASH, HCC. Higher levels are associated with a poor prognosis.
Platelet endothelial cell adhesion molecule 1 (PECAM-1 or CD 31) [147,148]	The higher the expression, the more severe or advanced the disease state; conversely, the lower the expression, the closer it is to a normal state.
MMPs and TIMPs [25,81,123]	MMPs’ values increase in fibrosis while TIMPs’ values decrease They better correlate with cirrhosis.
CK18 [77,135]	It has shown an AUROC of 0.84 for fibrosis detection in ALD patients.
Golgi protein-73 (GP-73) [135]	It has shown better correlation with cirrhosis in different CLDs with AUROC = 0.9.
Ten-eleven translocation protein 3 (TET3) [77,135]	Its values are higher in fibrosis patients. Its power is promising if combined with FIB-4.
Ferritin + BMI [77]	In NAFLD/MASLD patients, they can detect advance fibrosis and cirrhosis with AUROC = 0.87.
Oxidative stress biomarkers (MDA and SOD) [77]	In HCV infection, it has shown an AUROC of 0.9 for detecting fibrosis and an AUROC of 0.8 for detecting cirrhosis.
IFN-L3 [77]	It has been studied in HCV infection and a higher concentration in advanced fibrosis was revealed.
MFAP-4 [77]	It has an AUROC of 0.76 for detecting cirrhosis and an AUROC of 0.76 for detecting fibrosis.
α2m/hemopexin ratio [77]	In HCV Infection, it has shown an AUROC of 0.80 when correlated with significant fibrosis and an AUROC of 0.92 when correlated with advanced fibrosis.
sH2a + ALT [77]	In HCV infection, values yielded an AUROC of 0.79 for significant fibrosis detection and an AUROC of 0.86 for advanced fibrosis and cirrhosis detection.
AngioScore (AS) [37,39,145,146]	The AUC values were 0.886 for F.1, 0.920 for F.2, and 0.923 for F.3. A cutoff of 1.58 corresponded to a specificity of over 90%, identifying patients with low fibrosis and correctly classifying 67.5% of patients with an accuracy of 76.8%. The optimal cutoffs were more effective at identifying patients with moderate and severe fibrosis.

**Table 3:** Comparison between the methods of liver disease diagnosis/stratification.

Test	Pros	Cons
Liver biopsy (gold standard)	It represents a direct analysis of the histological status of the liver. This method is highly established, with the scoring system having been developed since 1980.	It is a surgical procedure that requires at least one day at the hospital, which means it can be expensive (it depends on national healthcare/health insurance). The procedure requires high expertise in sampling and interpreting results. The specimens represent only a minimal part of the entire liver, and often, they are unique. Results typically take about 2 weeks, depending on the laboratory efficiency. Standardization is largely dependent on manual processes. Patients are exposed to significant stress and substantial risks of pain, bleeding, and infections.
Imaging techniques	Usually, these refer to several techniques that allow the direct observation of the liver’s status and enable quantitative measures (CPA). These represent non-invasive, quick, and safe procedures. The standardization process for these techniques and imaging results is ongoing, and there is significant interest in using artificial intelligence algorithms for this purpose. Scores are based on the liver biopsy score system.	These procedures require high-cost instruments that are usually available only in specialized clinical settings. They are expensive for both the national healthcare system and for the patient. The failure rate and the effectiveness of the measures depend on the patient’s body characteristics, with higher failure rates in patients suffering from ascites and obesity. The expertise of the operators remains a key factor. Despite standardization efforts, measurements from different techniques are not comparable.
Serum biomarkers	Non-invasive, repeatable, cost-effective, safer, quick, and better-tolerated measurements. They enable early detection for at-risk patients and open the way to the development of new stratification and prognostication models. They rely on the detection of biomarkers that reflect the following: (I) changes in the ECM structure; (II) molecules derived from liver damage; (III) molecules related to liver function; (IV) molecules derived from the immune response after liver injury; (V) antibodies, antigens, nucleic acids of hepatotropic viruses; (VI) molecules impacting on liver metabolism; (VII) cytokines. Combining these biomarkers with imaging tests can positively impact results. These tests are usually well standardized, and the techniques are continually being developed.	Non-invasive tests are very promising but show high susceptibility to factors such as gender, age, sex, time of blood sampling, quality of blood sampling, and quality and modality of storage. Moreover, their specificity could be invalidated by non-liver inflammation. Single-marker measurements appear to be inefficient. The combination of different biomarkers or with imaging tests is instrumental in increasing accuracy. Importantly, this process can result in freely available formulas or proprietary (paid) algorithms Despite the general availability of routine laboratory tests, not all biomarkers are accessible in every facility, resulting in longer testing times. The more recent biomarkers require further study to be applied effectively and focused expertise to ensure the best result. Results should be carefully evaluated to understand their real meaning based on the stage of the disease.

## Data Availability

Data sharing is not applicable to this article, as no datasets were generated or analyzed during the current study.
